# Integrated Valorization of *Fucus spiralis* Alga: Polysaccharides and Bioactives for Edible Films and Residues as Biostimulants

**DOI:** 10.3390/foods13182938

**Published:** 2024-09-17

**Authors:** Valter F. R. Martins, Marta Coelho, Manuela Machado, Eduardo Costa, Ana M. Gomes, Fátima Poças, Raul A. Sperotto, Elena Rosa-Martinez, Marta Vasconcelos, Manuela E. Pintado, Rui M. S. C. Morais, Alcina M. M. B. Morais

**Affiliations:** 1 CBQF—Centro de Biotecnologia e Química Fina—Laboratório Associado, Escola Superior de Biotecnologia, Universidade Católica Portuguesa, Rua Diogo Botelho, 1327, 4169-005 Porto, Portugal; s-vfrmartins@ucp.pt (V.F.R.M.); mcoelho@ucp.pt (M.C.); mmachado@ucp.pt (M.M.); emcosta@ucp.pt (E.C.); amgomes@ucp.pt (A.M.G.); fpocas@ucp.pt (F.P.); emartinez@ucp.pt (E.R.-M.); mvasconcelos@ucp.pt (M.V.); mpintado@ucp.pt (M.E.P.); rcmorais@ucp.pt (R.M.S.C.M.); 2 Graduate Program in Plant Physiology, Botany Department, Biology Institute, Federal University of Pelotas, Pelotas 96160-000, Brazil; raulsperotto@gmail.com

**Keywords:** *Fucus spiralis*, edible films, bioactive compounds, polysaccharides, total phenolic content, antioxidant activity, cytotoxicity, biostimulant, germination, plant growth

## Abstract

*Fucus* spp. seaweeds thrive in the cold temperate waters of the northern hemisphere, specifically in the littoral and sublittoral regions along rocky shorelines. Moreover, they are known to be a rich source of bioactive compounds. This study explored the valorization of *Fucus spiralis* through the extraction of bioactives and polysaccharides (PSs) for food applications and biostimulant use. The bioactives were extracted using microwave hydrodiffusion and gravity (MHG), where the condition of 300 W for 20 min resulted in the highest total phenolic content and antioxidant activity of the extract. Cellular assays confirmed that the extract, at 0.5 mg/mL, was non-cytotoxic to HaCat cells. Polysaccharides (PSs) were extracted from the remaining biomass. The residue from this second extraction contained 1.5% protein and 13.35% carbohydrates. Additionally, the free amino acids and minerals profiles of both solid residues were determined. An edible film was formulated using alginate (2%), PS-rich *Fucus spiralis* extract (0.5%), and *F. spiralis* bioactive-rich extract (0.25%). The film demonstrated significant antioxidant properties, with ABTS and DPPH values of 221.460 ± 10.389 and 186.889 ± 36.062 µM TE/mg film, respectively. It also exhibited notable physical characteristics, including high water vapor permeability (11.15 ± 1.55 g.mm.m^−2^.day^−1^.kPa^−1^) and 100% water solubility. The residues from both extractions of *Fucus spiralis* exhibited biostimulant (BS) effects on seed germination and seedling growth. BSs with PSs enhanced pea germination by 48%, while BSs without PSs increased the root dry weight of rice and tomato by 53% and up to 176%, respectively, as well as the shoot dry weight by up to 38% and up to 74%, respectively. These findings underscore the potential of *Fucus spiralis* within the framework of a circular economy, wherein both extracted bioactives and post-extraction by-products can be used for sustainable agriculture and food applications.

## 1. Introduction

It is estimated that there are between 7500 to 10,000 species of seaweeds (marine macroalgae), which include green, brown, and predominantly red species [1]. Recently, macroalgae have gained popularity because they are a rich source of nutrients and they do not compete with food crops for land or water resources [2,3]. *Fucus spiralis* is a genus of brown algae found in the cold temperate waters of the littoral regions along the rocky shorelines of the northern hemisphere [1,2]. It is rich in bioactive compounds, such as phlorotannins, fucoxanthin, various vitamins, phenolic compounds, lipids, and polysaccharides, such as fucoidan [2]. It is associated with such health benefits as anti-inflammatory, antimicrobial, anticancer, antioxidant, anticoagulant, and antidiabetic properties [3,4,5,6,7]. According to the EU directive (EC 258/97), *Fucus vesicularis* was considered safe for human consumption. Fucoidan is used in coating and film formulations to preserve fruits and other food products [5,8].

Several authors have performed research aimed at enhancing the value of the algae *Fucus* spp. Anyanwu [9] highlighted the potential uses of algal biomass, including its application in biofuel production, as well as the utilization of its by-products in the fields of cosmetics, pharmaceuticals, wastewater treatment, and in food and feed. Other authors have developed edible films based on the whole biomass or extracts from algae, including *Fucus* spp. [10,11,12,13]. Trigo [10] produced a gelatine film with antimicrobial and antioxidant properties by adding lyophilized biomass from *Fucus spiralis*. Kajla [14] underlined the principal characteristics of edible films made from the seaweed polymers, carrageenan, agar, and alginate, such as their biodegradability, nontoxicity, antioxidant properties, and superior film-forming ability. Ganesan [15] obtained a sulphate polysaccharide from *Acanthophora spicifera* and produced an edible film for food applications. Perera [16] highlighted the ability of seaweed polysaccharides to produce active packaging, intelligent packaging, and edible films and coatings, despite several drawbacks, such as the low tensile strength, water solubility, and moderate antibacterial characteristics. Nevertheless, these limitations could be solved by adding other biopolymers, nanoparticles, or natural active agents.

Seaweeds and products derived therefrom are recognized for their potential as biostimulants in promoting plant growth [17]. Typically applied in the form of extracts, they stimulate various growth processes in plants and enhance their ability to resist stress by improving the efficiency of nutrient uptake and utilization [18]. While numerous studies detail the extraction of biologically active compounds from seaweeds to produce formulations applicable in agriculture [19,20,21,22], there is a notable lack of focus on the by-product: the post-extraction residue. However, this residue holds potential as a raw material for various industrial applications [23,24]. Managing this waste aligns with the principles of the circular economy, a crucial aspect of sustainable agriculture.

The present study aims at the integrated valorization of a *Fucus spiralis* through the extraction of bioactives and polysaccharides (PSs) for food applications and the biostimulant use for agricultural practices. Specific objectives are to extract bioactives from *F. spiralis* using microwave hydrodiffusion and gravity (MHG), to extract polysaccharides (PSs) from the remaining biomass and to make an edible film using the PS-rich *F. spiralis* extract and *F. spiralis* bioactive-rich extract, and to characterize this film in terms of several physicochemical properties. Another objective was to explore the potential of the residues from both extractions of *F. spiralis* as biostimulant (BS) on the seed germination and seedling growth of specific food crops. 

## 2. Materials and Methods

**Figure 1:**
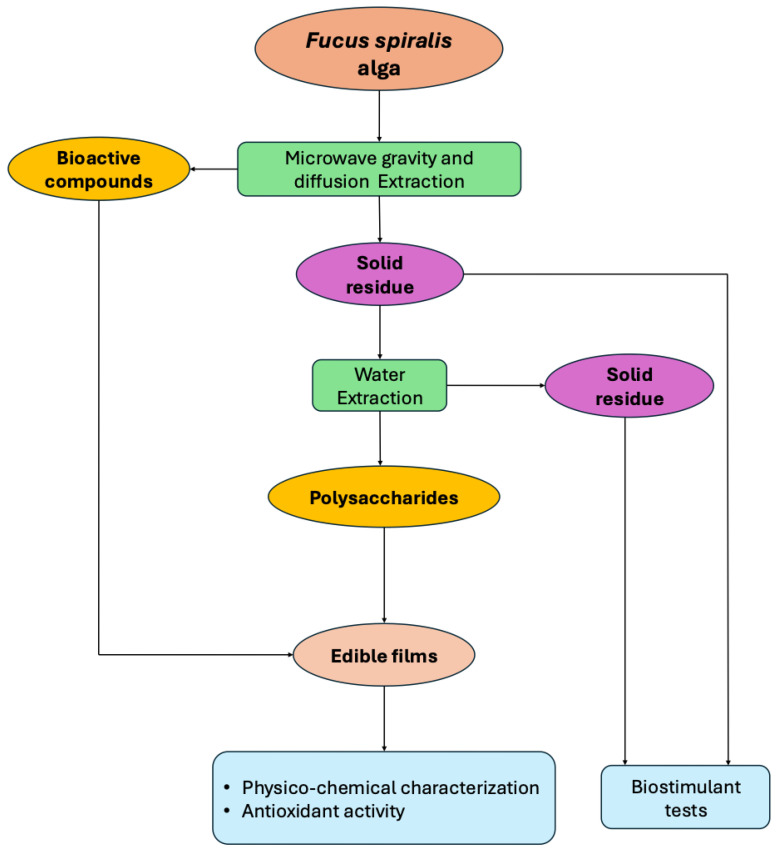
Overview of the experimental procedure for the integral valorization of the *Fucus spiralis* alga.

In this work, the integral valorization of the alga *Fucus spiralis* was performed according to Figure 1. It started with a green extraction of bioactives (extract rich in phenolics) by microwave hydrodiffusion and gravity (MHG), obtaining a first residue. This was re-extracted, obtaining a crude extract rich in polysaccharides (PSs) and giving place to a second residue. An edible film was made using a combination of the second extract (rich in PSs) and the first extract (rich in phenolics), and it was further characterized and assessed for its antioxidant capacity. The first and second residues (with and without PSs, respectively) were characterized in terms of the nutritional composition (only the second residue) and the free amino acids and minerals profiles, and these residues were subsequently tested as fertilizers (biostimulants) for the cultivation of three different crops.

### 2.1. Plant Material

*Fucus spiralis* alga was collected from a beach called “Memória” located in the region between Vila do Conde and Matosinhos, Portugal, on the 12 October 2022. It was quickly transported to the laboratory where it was cleaned with water, identified based on its morphological characteristics. The identification was performed by Prof. Dr. Isabel Sousa Pinto and an exemplar was kept at CIIMAR (Porto, Portugal). Subsequently, samples of 200 g were vacuum-sealed, frozen, and stored at −20 °C. For analysis, each sample was defrosted overnight at 10 °C.

### 2.2. Extraction of Bioactive Compounds 

*Fucus spiralis* processing was conducted in an advanced microwave extractor (Ethos, Milestone S.r.l., Sorisole, Italy) fitted with a vessel (1.5 L) with the capacity for 200 g of seaweed. Different operating conditions (power and time) were selected in order to irradiate the seaweed placed in the extractor operating at 50 Hz, and the resulting extracts were recovered by gravity. The extraction conditions were selected based on Perez et al.’s [25] and Florez-Fernandez et al.’s [26] research works. Three treatment conditions were selected: 300 W, 20 min (A); 300 W, 30 min (B); and 500 W, 20 min (C). The recovered liquid phase (extract) was stored at 4 °C until further analysis or until freeze-drying. The extraction yield was calculated according to Equation (1). The solid phase (first residue) was stored in the dark (−20 °C) for subsequent extraction of the polysaccharides.

Yield extraction (%) = weight of the extract × 100/weight of the alga (1)

### 2.3. Identification of Phenolic Compounds

The identification of the phenolic compounds in the *Fucus spiralis* extract was carried out by liquid chromatography electrospray ionization quadrupole time-of-flight mass spectrometry (LC-ESI-QqTOF-HRMS) following the methodology described by Vilas-Boas et al. [27] with some modifications. The extract was prepared by dissolving it in ultrapure water (50 mg/mL) and adding 1 mL of ice-cold MeOH (−80 °C) to precipitate the proteins. The separation was performed in a UHPLC UltiMate 3000 Dionex (Thermo Fisher Scientific Inc., Waltham, MA, USA), coupled to an ultra-high-resolution Qq-time-of-flight (UHR-QqTOF) mass spectrometer with 50,000 full-sensitivity resolution (FSR) (Impact II, Bruker Daltonics, Bremen, Germany), using an Acclaim RSLC 120 C18 column (100 mm × 2.1 mm, 2.2 µm) (Thermo Fisher Scientific Inc., Sunnyvale, CA, USA). The identification was based on standard solution retention time and mass spectra, when available, and the other peaks were tentatively identified based on the literature, and their elemental composition was confirmed through the accurate mass measurements (within 5 mDa of the assigned elemental composition) and mSigma values of <20. One independent analysis was performed for each of the triplicate extracts. 

### 2.4. Bioactivity Determination of the Liquid-Phase Extracts

The total phenolic content and antioxidant activity (ABTS, DPPH, and ORAC) of the extracts with bioactive compounds (Section 2.2) were determined. For these determinations, each extract was lyophilized to obtain a dried solid extract. This dried extract (40 mg) was resuspended in 2 mL of distilled water (20 mg/mL). Three replicates were performed.

#### 2.4.1. Total Phenolic Content (TPC)

The TPC was determined by the Folin–Ciocalteu method using the procedure described by Martins et al. [28]. The absorbance was measured at 765 nm (Synergy H1, Biotek, Winooski, VT, USA) in a 96-well microplate (Sarstedt, Numbrecht, Germany). Gallic acid was used as a standard for the calibration curve and the results were expressed as milligrams equivalent of gallic acid per milligrams of extract dry weight (mg GAE/mg DW). Three independent analyses were performed for each of the triplicates.

#### 2.4.2. Antioxidant Activity (AA)

The AA of the extract solutions mentioned above (20 mg/mL) was determined using three different assays.

##### ABTS

The ABTS (2,2′-azinobis(3-ethylbenzothiazoline-6-sulphonic acid)) assay was performed as described by Martins et al. [28]. Trolox was used as a standard for the calibration and the results were expressed as µmol of Trolox equivalent/milligrams of extract dry weight (µmol TE/100 mg DW). Three independent analyses were performed for each of the triplicates.

##### DPPH 

The DPPH (2,2-diphenyl-1-picrylhydrazyl) assay was carried out according to the procedure described by Martins et al. [28]. Trolox was used as a standard for the calibration. The results were expressed as µmol of Trolox equivalent/100 milligrams of extract dry weight (µmol TE/100 mg DW). Three independent analyses were performed for each of the triplicates.

##### ORAC

The ORAC assay was performed as described by Martins et al. [28]. The results were expressed as µmol of Trolox equivalent/100 milligrams of extract dry weight (µmol TE/100 mg DW). Three independent analyses were performed in each of the triplicates.

### 2.5. Cellular Cytotoxicity Evaluation of the Liquid-Phase Extracts

The evaluation of the cytotoxicity was carried out on the liquid extracts obtained using the extraction conditions A and C. It was performed according to the ISO 10993-5:2009 Standard as previously described by Costa et al. [29]. To simulate the skin, the cell line Hacat (Cell Line Services, Appenheim, Dinamarca) was used. Briefly, the cells were grown until 80–90% confluence; they were then detached using Tryple Express (ThermoScientific, Waltham, MA, USA) and put in microplates of 96 wells at 1 × 104 cells/well. After 24 h, the culture medium was removed and replaced by medium supplemented with the samples (previously freeze-dried) at various concentrations. Dimethyl sulfoxide (DMSO) (Sigma, St. Louis, MO, USA) at 30% (*v*/*v*) was used as dead control and medium without sample was used as cell growth control. After 24 h of incubation, PrestoBlue (Thermofisher, Waltham, MA, USA) was added to all wells and the microplate was re-incubated for 1 h. After this time, the fluorescence (Ex: 560 nm; and Em: 590 nm) was measured using a microplate reader (Synergy H1, Biotek Instruments, Winooski, VT, USA). The results were expressed in percentage of metabolism inhibition relative to the positive control. All assays were performed in quadruplicate.

### 2.6. Extraction of the Polysaccharides (PSs) 

The solid-phase residues (Section 2.2) were dispersed in distilled water at 50 g/L and were then heated near to boiling point (90 °C) with magnetic stirring (500 rpm) (IKAlaboratechnic RCT basic, Staufen, Germany) for 60 min. The resulting solution was filtered through filter paper, and the whole process was repeated. Finally, the PSs were precipitated, by adding ethanol (until 50:50 water-to-ethanol ratio) followed by centrifugation (5000 rpm, 20 min), redissolution in distilled water, and freeze-drying [30,31]. The extraction yield was determined and this extract rich in PSs was used to prepare the edible films (Section 2.7). The solid residue (second residue) was stored at −20 °C for the nutritional analysis and the determination of the free amino acid and mineral profiles, and to be used in subsequent tests as a fertilizer (Section 2.11).

### 2.7. Film Elaboration Based on the PSs 

Films were elaborated using Gomaa et al.’s [5] methodology with some modifications. Sodium alginate 2% (*w*/*v*) (Sigma, Aldrich Chemie Gmbh. Steinheim, Germany) was dissolved in hot distilled water and, then, 0.5% of the extracted PSs (Section 2.6) was added until total dissolution and left stirring for 1 h. After that, 0.6% (*v*/*v*) diacetyllauroyl glycerol (Tokyo Chemical Industry, Toshima, Kita-ku, Tokyo, Japan) was added to the alginate and PS solution and the mixture was stirred for 1 h at room temperature (25 °C). The film solution with PSs and enriched with bioactive extract from *Fucus spiralis* was obtained by adding the bioactive-rich extract (0.25% *w*/*v*) to the solution under stirring until complete dissolution. To obtain the film, 20 mL of the solution was poured into a 10 cm-diameter plastic Petri dish and left to dry at 40 °C for 18 h in an oven (Memmert GmbH, Schwabach, Germany). Another film was also tested only with 1% PS extract, but it did not present good physicochemical characteristics, as it was too adhesive, which made it difficult to handle. Nevertheless, it was possible to test this film for antioxidant activity. 

#### 2.7.1. Film Physicochemical Characteristics Determination

##### Thickness

A digital thickness gauge (Adamel Lhomargy, Ivry-sur-Seine, France) was used to measure the films’ thickness. Three replicates were prepared for each sample and five thickness measurements (at random positions) were taken for each replicate [32].

##### Colour

The colour of each film was analyzed with a colorimeter Chroma Meter CR 400 (Konica Minolta Sensing, Osaka, Japan) calibrated with a white standard colour plate. Colour measurements were in the system L*, a*, and b* [33]. Hue and Chroma were determined using the following equations:

Hue = arctan(b*/a*)(2)

(3) $$\mathrm{Chroma}=\sqrt{{a^{*}}^{2}+{b^{*}}^{2}}$$

Due to the transparency of the alginate film, its colour parameters (control) were measured while placed on the white standard colour tile.

##### Water Vapor Permeability 

The water vapor transmission rate (WVTR) and the water vapor permeability (WVP) of the films were determined gravimetrically at 23 ± 3 °C and 50% relative humidity (RH) in accordance with the standard ASTM E-96 [34]. Briefly, this technique consisted in placing dried calcium carbonate in a capsule and closing the capsules with the film. The capsule was weighed twice a day. The WVTR (g/m^2^/day) and WVP (g mm/m^2^/day/kPa) were determined through a linear regression and applying the formulae:

WVTR = weight of water that passed through the film/(area × time) (4)

WVP = WVTR × thickness of the film/(water vapor saturation pressure × ΔRH) (5)

##### Solubility

The determination of the film solubility was performed following the regulation EU Nº10/2011 [35]. Briefly, five solutions were prepared to test the film migration (water, water-to-acetic acid 3%, and water-to-ethanol 1:9, 2:8, and 5:5 *v*/*v*). The films were maintained in the solution for 24 h, and the solubility was determined using the equation:

Solubility (%) = 100 − film weight after 24 h immersion/film initial weight × 100 (6)

#### 2.7.2. Film Antioxidant Activity 

The antioxidant activity of the films was determined by the 2,2′-azinobis (3-ethylbenzothiazoline-6-sulfonic acid) (ABTS), and the 2,2-diphenyl-1-picrylhydrazyl (DPPH) scavenging assays, according to Lopes et al. [36]. Briefly, the ABTS solution concentration was adjusted with water to an initial absorbance of 0.700 ± 0.020 at 734 nm (Synergy H1, Biotek, Winooski, VT, USA). The DPPH working solution (90 µM) was prepared with methanol in order that the absorbance reached 0.600 ± 0.100 at 515 nm. Each film was cut into 1 mg pieces, which were placed into test tubes and the ABTS or DPPH solution (2–8 mL) was pipetted into each tube, protected from light exposition and at room temperature (25 °C). The tubes with ABTS and DPPH were left to react for 6 min and 30 min, respectively, and then the absorbance was measured at 734 nm (ABTS) and 515 nm (DPPH). The results were expressed as µM Trolox equivalents per mg of film (TE µM/mg film). All analyses were performed in quadruplicate.

### 2.8. Nutritional Characterization of the Solid Residue after the PS Extraction 

The dry matter, moisture, ash, fat, protein, fibre, and carbohydrate contents, and the energetic value of the solid residue after the PS extraction were determined.

#### 2.8.1. Moisture Content 

About 1 g of sample was placed in a Petri dish inside an oven at 105 °C for 24 h and subsequently weighed [37]. Three replicates were performed. The moisture content was calculated through the following equation:

Moisture content (%) = (weight at time 0 − weight of dried sample)/weight 0 × 100 (7)

#### 2.8.2. Ash 

About 2.5 g of sample was incinerated in a muffle furnace at 550 °C ± 15 °C until constant weight [38]. 

#### 2.8.3. Fat 

About 10 g of sample was boiled in a beaker (in reflux conditions, with dilute hydrochloric acid 4 M for 30 min) and filtered. The fat was extracted for 4 h with petroleum ether in a Soxhlet extractor into a previously weighed round bottom flask. The solvent was evaporated in a rotary evaporator (Buchi R-210, Buchi Labortechnik AG, Flawil, Switzerland) and the residue was dried to constant weight at 102 ± 2 °C [38]. 

#### 2.8.4. Protein 

The protein content was determined using ISO 1871:2009 [39]. Briefly, about 0.5 g of sample was digested in a mineralization block (Kjeltec Foss), with concentrated sulfuric acid (96% *w*/*w*) in the presence of a catalyst. From the quantity of ammonia produced, the nitrogen content (N) was calculated. The protein was calculated using the following formula %protein = %N × 6.25. At the same time, a blank test determination was performed.

#### 2.8.5. Fibre 

Briefly, the methods used were AOAC 991.43 [40] and AOAC 985.29 [41] and the assay was performed in duplicate. About 1 g of sample was submitted to enzymatic digestion using 3 enzymes: α-amylase (30 min at 90 °C), followed by protease (30 min at 60 °C), and followed by amyloglucosidase (30 min at 60 °C). To obtain the insoluble and soluble fibres in the first filtration (using celite), the residue was cleaned with 10 mL of water at 70 °C, and, then, with 10 mL of EtOH 95% and 10 mL of acetone. To determine the soluble fibre, four volumes of EtOH pre-heated at 60 °C were added and the residue was washed with 15 mL of EtOH 75%, EtOH 95%, and acetone. This residue was dried and the protein and ash contents were determined, the remainder being soluble fibre, subtracting the weight of celite. The total fibre was calculated by adding the insoluble and soluble fibre contents.

#### 2.8.6. Carbohydrates 

The carbohydrates were determined by difference, using the formula:

Carbohydrates = 100 − (moisture + ash + fat + protein) (8)

#### 2.8.7. Determination of the Energetic Value

For the determination of the energetic value, the following formula (Regulation UE nº 1169) [42] was used:

Energetic value (kcal/100 g sample) = 4 × (mass_protein_ + mass_carbohydrates_) + 9 × mass_fat_ (9)

### 2.9. Determination of Amino Acid Profiles of the Solid Residues (Section 2.2 and Section 2.6)

#### 2.9.1. Sample Preparation 

A 10 mg sample was placed in a vial for solid-phase micro-extraction (SPME) and mixed with 3 mL of 6 M HCL (Sigma-Aldrich, Inc., St. Louis, MO, USA). The mixture was vortexed, bubbled with nitrogen for 4 min, then sealed with tape and incubated in an oven at 115 °C overnight. After cooling, 4 mL of MILLI-Q water was added, and the pH was adjusted to 3.5 with 10 M NaOH. The final volume was brought to 10 mL with MILLI-Q water, and the samples were filtered with a 0.45 μm filter. 

#### 2.9.2. HPLC Analysis

Qualitative and quantitative profiles of amino acids were carried out according to the method proposed by Long [43]. Briefly, the reagents involved were mixed with 100 µL of filtered sample (10 mg/mL of freeze-dried hydrolysate), and 10 µL of the mixture was injected into the HPLC. After preparation (Section 2.9.1), the samples were analyzed using the Agilent amino acid HPLC method developed based on Agilent Poroshell HPH-C18 Column (2.1 × 200 mm, 5 μm, Agilent, Santa Clara, California, USA) as described by Long [43]. The analysis was performed using an Agilent 1200 series HPLC system consisting of an LC-20AB solvent delivery unit, a SIL-20A autosampler, a CTO-20A column oven (25 °C), a G1321A FLD (excitation at 356 nm; emission at 4445 nm), and an LC Ver1.23 workstation (Agilent Technologies). The separation process used a Hypersil AA-ODS column from Agilent Technologies. Mobile phase A consisted of 10 mM Na_2_HPO_4_, 10 mM Na_2_B_4_O_7_ (pH 8.2), and 5 mM NaN_3_ (Sigma-Aldrich, Inc., St. Louis, MO, USA) with pH adjusted to 8.4 using concentrated HCl. It was filtered through 0.45 µm regenerated cellulose membranes (p/n 3150-0576). Mobile phase B was a mixture of acetonitrile (Fisher Chemical, Pittsburgh, Pennsylvania, USA), methanol (Fisher Chemical), and water (45:45:10). The elution conditions involved a linear gradient from 90% A to 50% A over 43 min, followed by a steady increase of B up to 100% B from 47 to 49 min, and returning to 90% by 50 min using a flow rate 0.9 mL/min. Standards for alanine, asparagine, aspartic acid, cysteine, glutamic acid, glutamine, glycine, isoleucine, leucine, methionine, valine, proline, serine, threonine, phenylalanine, tryptophan, and tyrosine were prepared at 100 mg/L, sourced from Sigma Chemical Co. (St. Louis, MO, USA).

### 2.10. Determination of the Mineral/Oligoelement Profiles of the Solid Residues 

The mineral profile was determined according to the method described in the compendium for plant tissue [44]. After weighing 0.5 g of the sample into the digestion vessel, 10 mL of nitric acid was added. The mixture was gently swirled for approximately 15 min and, thereafter, placed in the microwave extraction (Mars One, Matthews, North Carolina, USA) vessel under the digestion conditions of 200 °C and 800 psi. Once the extract was obtained, the volume was made up of 50 mL MILLI-Q water. The concentration of minerals was measured by ICP-MS (BRUKER Aurora-M90 ICP-MS), according to the method OIV-MA-ASS 322, EN 13805, and EN 14084 [44], in duplicate. All standard curves used—for the following elements Zn, Fe, Cu, Na, K, Ca, and Mg (all purchased from Sigma Chemical Co. (St. Louis, MO, USA)—were prepared at 100 mg/L.

### 2.11. Plant Cultivation with Fucus Spiralis Residues

The residues of *Fucus spiralis* without (−) or with (+) PSs were lyophilized, homogenized with liquid nitrogen, and mixed with a substrate (COMPO SANA, a mixture of peat, perlite, agrosil, guano, and NPK fertilizer; pH 6.0; 97% organic matter) at two different concentrations (0.05% and 0.5% *w*/*w*) to test whether they could exert a biostimulant (BS) effect on the germination of pea, rice, and tomato seeds, as well as on the initial growth of these plants [19]. Therefore, the experiment consisted of five treatments: Control (no BS added); BS-PS 0.05%; BS-PS 0.5%; BS+PS 0.05%; and BS+PS 0.5%. Plastic trays with small cells were filled with these substrates, and pea (*Pisum sativum* L. cv. Tom Thumb), rice (*Oryza sativa* L. cv. Nipponbare), and tomato (*Solanum lycopersicum* L. cv. Coração de Boi) seeds were germinated at a rate of one seed per cell. The seedlings were irrigated according to the plants’ needs. Plants were germinated and grown in a climatic chamber set at 25 °C and 60% moisture, with a photoperiod of 16 h light and 8 h dark. After 14 days, the germination rate of each species was assessed (*n* = 6 for pea and tomato; *n* = 3 for rice) [19]. At 14, 22, and 28 days after sowing (DAS), the pea, rice, and tomato seedlings, respectively, were removed from the tray, the roots were carefully washed, the roots and shoots were separated, the length of the roots and shoots were measured (*n* = at least 18), and they were dried separately for 3–4 days. After complete drying, the dry weight of the roots and shoots were obtained (*n* = at least 18) [17].

### 2.12. Statistical Analysis

The results were expressed as mean ± standard deviation of three independent replicates (n = 3). Shapiro–Wilk (for normality) and Levene (for homogeneity of variance) tests were performed on the residuals of the fitted model. All data demonstrated normal distribution and were statistically compared (control vs. different formulations) using one-way ANOVA, followed by Tukey’s test (*p* ≤ 0.05) in the case of homogeneous variance, or followed by Dunnett’s C test (*p* ≤ 0.05) in the case of heterogeneous variance. Student’s *t*-test was used to detect significant differences between two groups of results. SPSS Base 23.0 for Windows (SPSS Inc., Armonk, USA) was used for statistical analysis.

## 3. Results and Discussion

### 3.1. Extraction of Bioactive Compounds and Identification of the Phenolic Compounds in the Liquid-Phase Extracts 

The extraction yield was 48.99 ± 3.23% for the liquid extract and 0.278 ± 0.042% for the freeze-dried extract.

In the genus *Fucus,* it is common to find compounds of the phlorotannins class, which is very complex and whose extracts reveal a wide spectrum of compounds, such as phloroglucinol, fucols, fuhalols, and several fucophlorethol structures with different degrees of polymerization, and these compounds normally have high molecular weights [8,45,46,47]. 

In the present study, the MHG extract analyzed by LC-ESI-QqTOF-HRMS presented three compounds that are reported in literature: a phlorotannin derivative (peak 4 presents a [M-H]^−^ at *m*/*z* 611.12), an unknown compound (peak 7 presents a [M-H]^−^ at *m*/*z* 223.08), and difucol (peak 12 presents a [M-H]^−^ at *m*/*z* 249.10). The remaining compounds are unknown (Table 1). 

The chromatographic analysis showed that most of the compounds present in the *Fucus spiralis* MHG extract correspond to peaks 10, 6, 9, 12, and 1. The other peaks are not significant. There was a high variability observed among different algal extracts concerning the phlorotannin composition. This may be due to the influence of specific factors, such as thallus age and physiological variations within algal organs, or to extrinsic factors (origin, time of harvest, and general surrounding conditions) [46]. 

Phlorotannins have a wide range of biological activities, such as antioxidant, antibacterial, antiviral, anti-tumour, antihypertensive, hypoglycemic, whitening, anti-allergic, and anti-inflammatory activities. These compounds may be used in the food area [48].

### 3.2. Bioactivity of the Liquid-Phase Extracts

Both Table 2 and Table 3 show that the highest values of TPC and AA (ABTS, DPPH, and ORAC) correspond to the extraction performed using the MHG conditions 300 W and 20 min. The liquid extract obtained from this extraction condition and containing phenolic compounds was selected for the film production.

Lopez-Horta et al. [49] found similar results: a higher TPC and AA (ABTS) using MHG at 300 W to extract bioactive compounds from *Undaria pinnatifida* and *Laminaria ochroleuca* seaweeds. Heffernan et al. [50] prepared an extract rich in phlorotannins using water and making a dialysis. They determined the values for TPC in phloroglucinol equivalents (μg PE/mg sample) of 231.95 ± 8.97 in *Fucus vesiculosus* and 180.55 ± 16.98 in *Fucus serratus*, and the values for DPPH IC_50_ (mg/mL) of 4.00 ± 0.01 in *Fucus vesiculosus* and 19.00 ± 0.03 in *Fucus serratus*.

### 3.3. Cellular Assays for Cytotoxicity Evaluation

**Figure 2:**
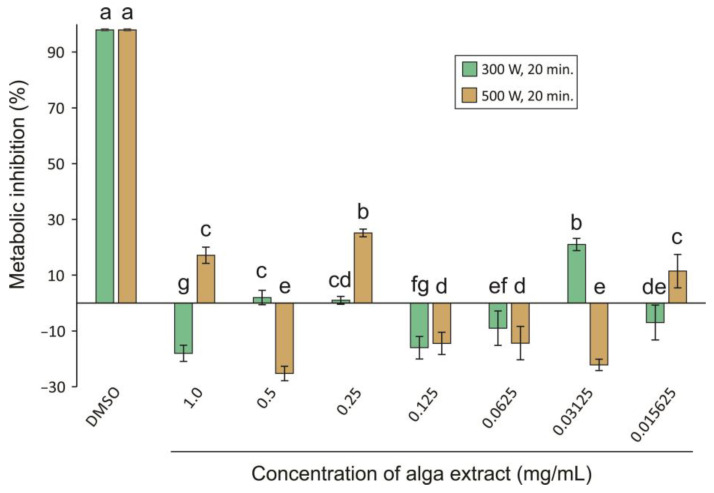
Cytotoxicity (% metabolism inhibition) of samples of alga extract using MHG condition 300 W, 20 min, and condition 500 W, 20 min in HaCat cell line. Different letters mean significant differences (*p* < 0.05) among different concentrations of the extract.

The cytotoxicity assay was performed using the HaCat cell line on the extracts from MHG conditions 300 W, 20 min and conditions 500 W, 20 min. The results obtained are depicted in Figure 2, and show that, at the concentrations tested (from 0.015625 to 1 mg/mL), neither of the samples showed any cytotoxicity in relation to the cells used. Tukey’s test was used to compare the results obtained for different concentrations in each extraction condition (in Figure 2, different letters mean significant differences among different concentrations of the extract). Student’s *t*-test revealed that the results between extracts for each concentration were significantly different, including for 0.5 mg/mL.

*Fucus spiralis* is one of the 22 species of seaweed that are considered safe to be consumed under the European directive (EC 258/97), which means that it is not cytotoxic. André et al. [51] tested a Caco-2 cellular line that simulated the intestinal barrier, precisely, the human colorectal adenocarcinoma epithelial cell line. He observed that a *F. vesiculosus* extract rich in phlorotannins at concentration of 0.25 mg/mL did not show cytotoxicity for the cell line studied and showed a reduction of 45.3 ± 4.4% in the cholesterol permeation. Catarino et al. [46] concluded that this alga sample presented cytotoxicity against tumour cell lines, namely, Caco-2 colorectal and MKN-28 gastric cancer cells, without affecting normal cells.

### 3.4. Extraction Yield of the PS

The dried weight of the alga was 16.78 ± 0.75% (83.22% water), and the extraction yield of the PSs was 1.315 ± 0.255% fresh weight and 7.841 ± 0.255% dry weight. Zaim et al. [31] studied brown algae and obtained 13% extracted PSs and 85.05% water for *Sargassum meticum*, 15.27% extracted PSs, and 82.53% water for *Cystoseira myriophylloides*, and 15.09% extracted PSs and 84.13% water for *Cystoseira baccata*. All these yield values were higher than the one found in the present study for *Fucus spiralis.* The water content was similar.

### 3.5. Film Based on the PSs

#### 3.5.1. Physicochemical Characteristics of the Films

##### Thickness

The thickness of the film with alginate (2%), *Fucus spiralis* PSs (0.5%), and *Fucus spiralis* bioactive-rich extract (0.25%) was 0.127 ± 0.004 mm, higher than the value obtained by Gomaa et al. [5], which was 0.083 ± 0.009 mm, for a film containing alginate and fucoidan from *Sargassum latifolium*.

##### Colour

**Figure 3:**
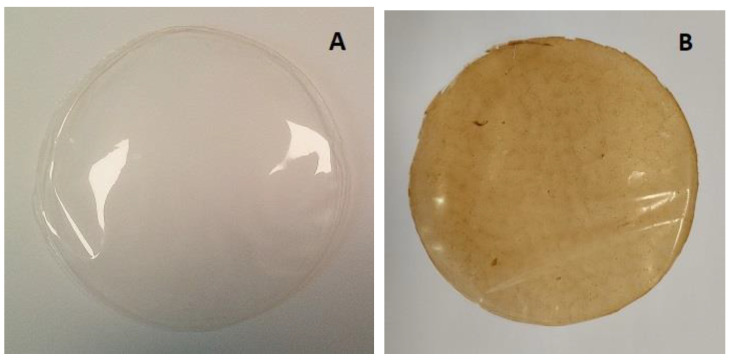
Film with (**A**) alginate (3%) and (**B**) alginate (2%) + *Fucus spiralis* PSs (0.5%) + *Fucus spiralis* bioactive-rich extract (0.25%).

The film obtained had a yellow-brown colour (Figure 3). The values of L* (lightness), a* (redness/greenness), and b* (yellowness/blueness) are generally low (Table 4). With the exception of the L* parameter, all other colour parameters presented significant differences between both films. Gomaa et al. [5] measured the colour of alginate films with polysaccharides including fucoidan from *Sargassum latifolium* (brown) and obtained the values L* = 58.34 ± 3.93, a* = 11.61 ± 2.85, and b* = 58.82 ± 3.93, higher than those of the films of the present work.

##### Water Vapor Permeability 

The water vapor permeability (WVP) and water vapor transmission rate (WVTR) for the alginate (2%) + *Fucus spiralis* PSs (0.5%) + *Fucus spiralis* bioactive-rich extract (0.25%) film was 11.15 ± 1.55 g.mm.m^−2^.day^−1^.kPa^−1^ and 127.11 ± 17.67 g.m^−2^.day^−1^, respectively. These values are higher than those reported by Gomaa et al. [5], who obtained WVP = 4.24 ± 0.14 × 10^−10^ g/(m.s.Pa) and WVTR = 36.6 ± 1.2 g.m^−2^.day^−1^ for an alginate film with fucoidan from *Sargassum latifolium*. 

##### Solubility 

The alginate (2%) + *Fucus spiralis* PSs (0.5%) + *Fucus spiralis* bioactive-rich extract (0.25%) film has a higher or complete solubility in water and hydroethanolic solutions at 10 and 20% (simulating hydrophilic foods [35]), and moderate solubility in an hydroethanolic solution at 50% (simulating lipophilic foods [35]) and with a water-to-acetic acid ratio of 3% (simulating foods with pH lower than 4.5 [35]) (Table 5). There was a significant difference in the solubility between the film with *Fucus spiralis* PSs and the control (3% alginate) only for the hydroethanolic solution at 50%.

Gomaa et al. [5] found a similar result, 94.40%, of solubility in water after 24 h for an alginate film with fucoidan from *Sargassum latifolium*. 

#### 3.5.2. Antioxidant Activity (ABTS and DPPH) of the Films

As expected, the values obtained with the ABTS method are higher than the ones with the DPPH method (Table 6) given that, in the ABTS method, both hydrophilic and lipophilic compounds are quantified, whereas, in the DPPH method, only lipophilic compounds are tested. 

The film with alginate, *Fucus spiralis* PS extract (0.5%), and the bioactive-rich extract (0.25%) had higher values for ABTS and DPPH than the control film (alginate 3%). A film with the formulation with the *Fucus spiralis* PS extract (1%) plus the *Fucus spiralis* bioactive-rich extract (0.25%) was tested, but it was too adhesive, being difficult to handle, and the results for the ABTS and DPPH assays were 428.357 ± 35.839 and 423.439 ± 80.242 µM TE/mg film, respectively. This shows that the *Fucus spiralis* PS extract has a high antioxidant activity, and this increased with the *Fucus spiralis* PS concentration in the film. 

Gomaa et al. [5] studied the antioxidant activity of an alginate and fucoidan film and obtained a total antioxidant capacity (TAC) of 0.48 ± 0.051 g ascorbic acid equivalent/g film, a ferric reducing antioxidant power (FRAP) of 3.47 ± 0.12 mg ascorbic acid equivalent/g film, and a hydroxyl radical scavenging activity (HRSA) of 84.92 ± 2.4%.

### 3.6. Nutritional Characterization of the Solid Residue after PS Extraction

The nutritional composition of the residue resulting from the PS extraction of *Fucus spiralis* is in Table 7. As it provides a comparison of these results for *Fucus spiralis* with those obtained by Catarino et al. [2] for *Fucus* spp., it is possible to observe that only the ash and carbohydrate contents of the residue were below the results found in literature. This has an influence on the energetic value of the alga residue that is significantly lower than the algae analyzed by Catarino et al. [2].

Nevertheless, it is necessary to consider that the results may differ according to the method used. For example, for the protein content, the Kjeldahl method can give values much different from those obtained with HPLC, because, during the sample digestion, some nitrogen can be lost [52,53].

### 3.7. Determination of the Amino Acid Profiles of the Residues from the MHG and PS Extractions

In general, the values of protein in this alga *Fucus spiralis* are low, the higher values being found at the end of winter or spring [2]. Different amino acids have different functions in the human organism. For example, aspartic acid and glycine are responsible for the formation of new tissues and regulation of the nervous system, while lysin and isoleucine are important for the immunologic system and phenylalanine for the thyroid function [2].

The contents in amino acids of both residues (with and without PSs) are found in Table 8. In general, both residues (with and without PSs) presented lower values when compared with the literature for each quantified amino acid. The most abundant amino acids in the analyzed residues are histidine and glycine, while, in literature [2], the most abundant are glutamic acid and asparagine.

Histidine, leucine, isoleucine, lysine, valine, methionine, phenylalanine, tryptophan, and threonine are the nine essential amino acids crucial for underlining the protein biological value both in terms of nutritional quality and bioavailability. Histidine, valine, leucine, methionine, and threonine are present in both algae residues in a total of 15.98 and 33.5 mg/100 mg DW, respectively; these values are aligned with those reported in literature, in particular in the PS-free residue [54]. 

The comparative analysis of free amino acid concentrations in algae residues with and without polysaccharides reveals that the removal of polysaccharides generally results in significantly higher amino acid concentrations. Besides the concentration effect, this may indicate that the high amount of cell wall anionic polysaccharides may bind or otherwise limit the availability of free amino acids in algae residues [52,55,56]. Notably, aspartic acid, cysteine, asparagine, histidine, glycine, and valine exhibit substantial increases in their concentrations when polysaccharides are absent, with histidine showing the most marked increase from 11.49 mg/100 mg DW to 22.92 mg/100 mg DW. Conversely, alanine and the essential amino acid tryptophan were not detected in either condition, which can be due to protein degradation during the extraction procedures.

The comparison with *Fucus* spp. indicates that several amino acids, such as histidine, glycine, and valine, in the polysaccharide-free residues, align closely with the ranges reported for *Fucus* spp. [2,57,58]. 

These findings suggest that algae residues, especially those without polysaccharides, could serve as a rich source of free amino acids, holding potential for various nutritional and industrial applications.

### 3.8. Determination of the Mineral/Oligoelement Profile of the Solid Residue from the PS Extraction

The content in minerals is associated with the polysaccharides because these compounds retain inorganic marine substances. Nevertheless, the mineral content of seaweeds is very variable according to the geographic harvesting site, wave exposure, and seasonality [59].

The mineral contents in both residues (with and without PSs) are shown in Table 9. Comparing the results of mineral contents in the two residues (with and without PSs), it is possible to observe that the residues have a mineral content below the minimum present in the *Fucus* spp. in the data collected by Catarino et al. [2]. One explanation for this fact could be that the minerals and oligoelements were lost during the process of PS extraction. Nevertheless, it is possible to observe that all mineral contents were higher after the MHG extraction (first column) than after the PS extraction (second column) (Table 9). The most abundant minerals in these residues are sodium and potassium as reported in literature.

The presence of polysaccharides in algae residues appears to enhance the retention of certain minerals, such as zinc, iron, sodium, potassium, calcium, and magnesium. This indicates that polysaccharides might play a role in binding and stabilizing these minerals within the algae matrix. However, the mineral content in both algae residues with and without PSs is generally lower than the ranges reported for *Fucus* spp. This discrepancy could be attributed to differences in species, environmental conditions, and the specific processing methods used for the algae residues [54,58].

The absence of detectable copper in both types of algae residues suggests either a lower natural abundance or potential loss during the extraction and analysis processes. This contrasts with the detectable levels of copper in *Fucus* spp. [2].

### 3.9. Fucus Spiralis Residue as a Plant Biostimulant

**Figure 4:**
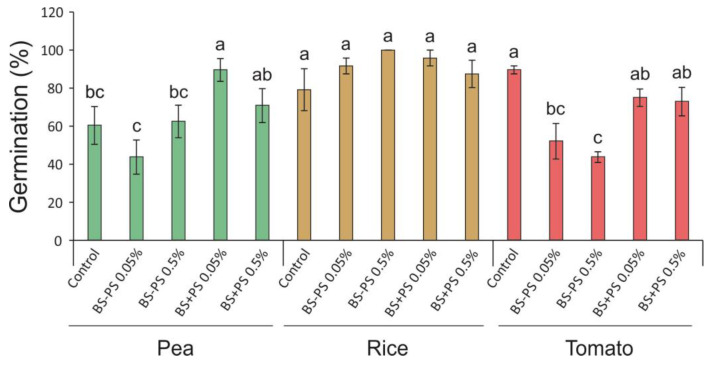
Germination rate (%) of pea, rice, and tomato seeds at 14 days after sowing treated with *Fucus spiralis* residue (BS = biostimulant) with (+) or without (−) polysaccharides (PSs) in two different concentrations (0.05% and 0.5%). Bars represent mean ± SE (n = 6 for pea and tomato; and n = 3 for rice). Different letters indicate statistically significant differences (*p* < 0.05) between tested conditions (Control, BS-PS 0.05%, BS-PS 0.5%, BS+PS 0.05%, and BS+PS 0.5%) in each plant species.

Fourteen days after sowing (DAS), no effect of the *Fucus spiralis* residue was detected in rice germination. At the same time, BS+PS 0.05% increased pea germination by 48% compared to the control condition, while both BS-PS treatments (0.05% and 0.5%) decreased tomato germination by 42% and 51%, respectively (Figure 4).

The reasons for the stimulatory effect of the *Fucus spiralis* residue with PS specifically on pea seed germination would largely depend on the PS characteristics. Similarly, further research would be necessary in order to elucidate the underlying mechanisms controlling the inhibitory effect of the *Fucus spiralis* residue without PS specifically on tomato seed germination. Sivritepe and Sivritepe [60] analyzed the germination of pepper after priming seeds in a 10% seaweed extract of *Ascophyllum nodosum* at dilutions of up to 1:1000. A distinct suppression of the germination rate was evident with the seaweed extract at concentrations of 1:250 and above compared to control seeds primed solely with water. The results indicate no enhancement in germination rates with the less concentrated extracts compared to their water counterparts. According to Reitz and Trumble [61], the level of response displayed by a seaweed extract varies depending on the plant species and even the specific cultivar being cultivated. Furthermore, under particular conditions, an extract might even demonstrate inhibitory effects, with the minimal response to seaweed typically observed in plants cultivated under conditions close to optimal, as might be predicted [62]. Therefore, it would be interesting to test the effects of *Fucus spiralis* residues on seed germination (as well as seedling development) under non-optimal conditions.

**Figure 5:**
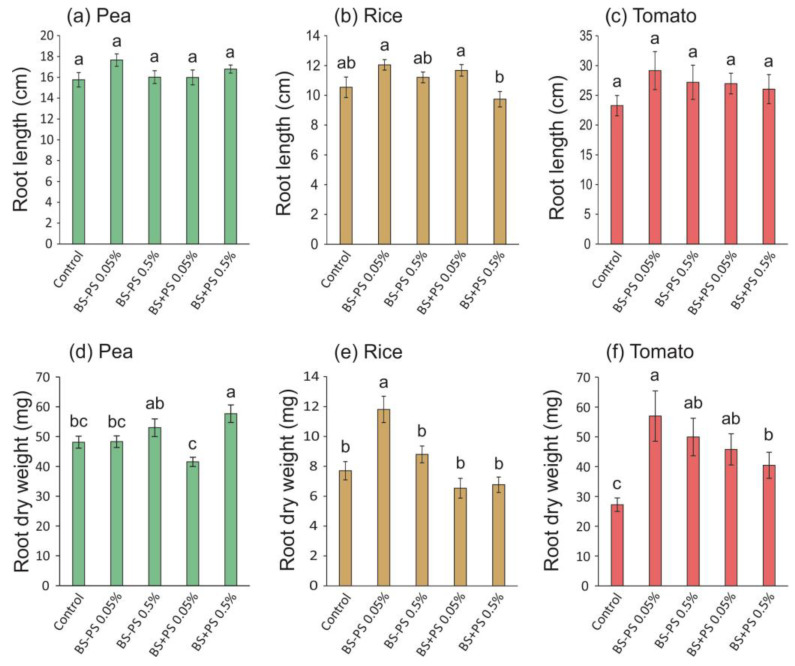
Root length and dry weight of pea (**a**,**d**), rice (**b**,**e**), and tomato (**c**,**f**) seedlings treated with *Fucus spiralis* residue (BS = biostimulant) with (+) or without (−) polysaccharides (PSs) in two different concentrations (0.05% and 0.5%) for 14 days (pea), 22 days (rice), and 28 days (tomato). Bars represent mean ± SE (n = at least 18). Different letters indicate statistically significant differences (*p* < 0.05) between tested conditions (Control, BS-PS 0.05%, BS-PS 0.5%, BS+PS 0.05%, and BS+PS 0.5%) in each plant species.

Comparing with the control condition, none of the treatments affected the root length, regardless of the plant species (Figure 5a–c). However, the root dry weight of all plant species was enhanced by *Fucus spiralis* residues, with a 20% increase in pea (BS+PS 0.5%, 14 DAS—Figure 5d), a 53% increase in rice (BS-PS 0.05%, 22 DAS—Figure 5e), and an up to 176% increase in tomato (BS-PS 0.05%, 28 DAS—Figure 5f). 

**Figure 6:**
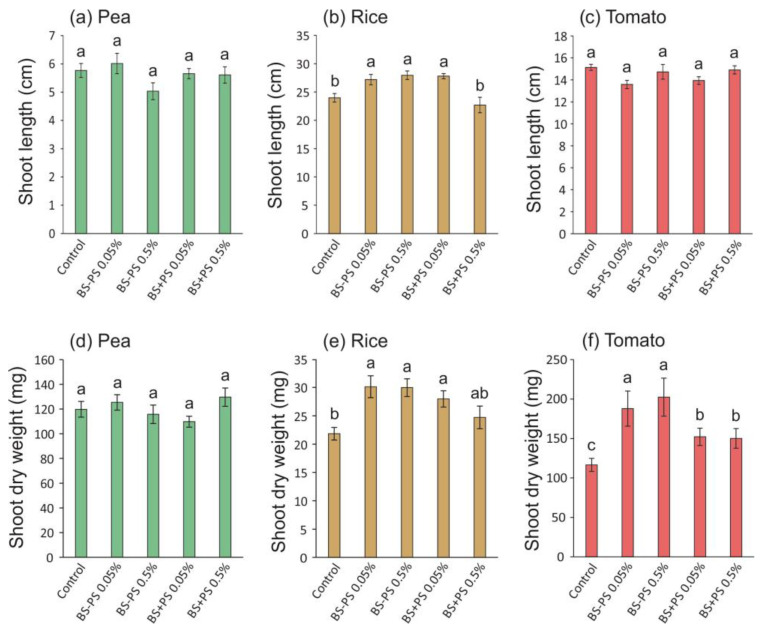
Shoot length and dry weight of pea (**a**,**d**), rice (**b**,**e**), and tomato (**c**,**f**) seedlings treated with *Fucus spiralis* residue (BS = biostimulant) with (+) or without (−) polysaccharides (PSs) in two different concentrations (0.05% and 0.5%) for 14 days (pea), 22 days (rice), and 28 days (tomato). Bars represent mean ± SE (n = at least 18). Different letters indicate statistically significant differences (*p* < 0.05) between tested conditions (Control, BS-PS 0.05%, BS-PS 0.5%, BS+PS 0.05%, and BS+PS 0.5%) in each plant species.

Regarding the shoots, a stimulatory effect of *Fucus spiralis* residues was only observed for rice (up to 17% increase in shoot length with BS-PS 0.5% treatment—Figure 6b; and up to 38% increase in shoot dry weight with BS-PS 0.05% treatment—Figure 6e) and tomato (up to 74% increase in shoot dry weight with BS-PS 0.5% treatment—Figure 6f). 

The use of seaweed extracts in crop management has become widespread owing to their documented abilities to enhance growth and resilience to environmental stresses [63,64,65,66]. Therefore, several works have demonstrated beneficial effects of seaweed extracts on the growth/yield of different plant species [19,20,21,22,64,67,68,69,70]. However, few works have described the beneficial effects of seaweed post-extraction residues, following the concepts of sustainable agriculture and a circular economy. Recently, Krautforst et al. [17] reported a comprehensive approach to the management of brown seaweed *Fucus vesiculosus*, which could be used to increase radish (*Raphanus sativus*) seed germination as an algal extract (20%), and could be used to increase sorghum (*Sorghum saccharatum*) plant growth in the initial phase as an algal biomass (2 g/kg of soil additive) and a post-extraction residue (2 g/kg of soil additive). Accordingly, Bikovens et al. [71] investigated the impact of soil amendments with the *Fucus vesiculosus* post-extraction residue (10 g/kg) on oat (*Avena sativa*) growth. The authors underscored the value of the post-extraction residue, containing residual biological and antioxidant compounds like fucoidan and phenolics, as a promising fertilizer additive in agricultural practices. As the most pronounced effects in our work were found in *Fucus spiralis* residues without polysaccharides (-PS; Figure 5 and Figure 6), it would be interesting to identify the remaining compounds after extraction that promote these biostimulant effects. We also believe that it would be interesting to test the effect of *Fucus spiralis* residues on the development of pea, rice, and tomato plants under different stressful conditions, as well as on the seed/fruit production and quality.

## 4. Conclusions

The extract rich in bioactive compounds extracted from *Fucus spiralis* using MHG showed good antioxidant capacity. It was possible to elaborate an edible film based on alginate and polysaccharides extracted from the residue resulting from that extraction. The film incorporated with the bioactive-rich extract also exhibited antioxidant activity, which increased with the PS concentration in the formulation. The film with 2% alginate + 0.5% *Fucus spiralis* PSs + 0.25% bioactive-rich extract was highly soluble in water, ethanolic solutions, and acetic acid, and, thus, was likely to be biodegradable. All studied aspects agreed with the possibility of using this film in the food industry.

The removal of polysaccharides from algae residues generally leads to increased concentrations of free amino acids. This trend is observed for most of the amino acids examined. Notably, many amino acids in the algae residues without polysaccharides meet or surpass the concentration ranges reported for *Fucus* spp., suggesting that these residues could be a valuable source of amino acids. Further investigation is needed to understand the mechanisms underlying polysaccharide–mineral interactions and their nutritional implications. Additionally, refining extraction processes could enhance the potential use of these residues in various applications, such as nutritional supplements or protein sources.

The nutritional composition and the amino acids and minerals profiles of the residues obtained in the whole process (two extractions used, one to obtain the bioactive-rich extract and another to obtain the PSs) suggest that these residues could be used as biofertilizers. Three plant species, pea, rice, and tomato, exhibited distinct responses to the application of *Fucus spiralis* residue as a biostimulant. The most promising outcomes were observed for pea during germination (+PS 0.05%), and for rice and tomato during early growth, particularly evidenced by a significant increase in the root (-PS 0.05%) and shoot dry weight (-PS 0.05% and 0.5%).

## Figures and Tables

**Table 1:** Tentative assignment of the compounds present in the aqueous fraction of *Fucus spiralis* extract obtained by MHG and analyzed by LC-ESI-QqTOF-HRMS.

Peak	RT (min)	[M-H]^−^ (*m*/*z*)	Molecular Formula	MS/MS Fragments (-loss)	m Sigma	Tentative Assignment	References
1	2.1	191	C_13_H_4_O_2_	191, 111	44.12	-	-
2	2.57	223	C_15_H_12_O_2_	223, 152, 96	45.2	-	-
3	6.2	243	C_10_H_12_O_7_	243, 150	3.8		
4	6.48	611	C_33_H_24_O_12_	611, 306, 227	∞	Phlorotannin derivative	[38]
5	30.41	347	C_18_H_20_O_7_	347, 145	22.54	-	.
6	32.18	209	C_7_H_14_O_7_	209, 141, 118	18.39	-	-
7	36.28	223	C_8_H_16_O_7_	223, 149	11.12	Unknown	[5]
8	37.58	225	C_8_H_18_O_7_	225, 149	19.39	-	-
9	38.05	253	C_10_H_22_O_7_	253, 207	18.83	-	-
10	38.61	253	C_10_H_22_O_7_	253, 207	15.71	-	-
11	39.26	223	C_8_H_16_O_7_	223, 149	32.77	-	-
12	39.44	249	C_10_H_18_O_7_	249, 149	22.88	Difucol	[39]

**Table 2:** Total phenolic content of the liquid-phase extracts obtained by MHG.

MHG Extraction Conditions	TPC (mg GAE/mL Extract)	TPC (mg GAE/100 g Alga)
300 W, 20 min	0.086 ± 0.015 ^a^	3.25 ± 0.97 ^a^
300 W, 30 min	0.043 ± 0.009 ^b^	3.23 ± 0.37 ^a^
500 W, 20 min	0.045 ± 0.004 ^b^	3.55 ± 0.19 ^a^

Different letters in each column mean statistically significant differences (*p* < 0.05).

**Table 3:** Antioxidant activity (ABTS, DPPH, and ORAC) of the extracts obtained by MHG.

MHG Extraction Conditions	ABTS	DPPH	ORAC
	(µmol TE/100 mg Sample DW)		
300 W, 20 min	3.530 ± 0.477 ^a^	0.900 ± 0.104 ^a^	4.385 ± 0.508 ^a^
300 W, 30 min	1.990 ± 0.297 ^b^	0.485 ± 0.090 ^b^	1.805 ± 0.606 ^b^
500 W, 20 min	1.790 ± 0.134 ^b^	0.468 ± 0.014 ^b^	1.149 ± 0.244 ^b^

Different letters in each column mean statistically significant differences (*p* < 0.05).

**Table 4:** Colour parameters of the alginate (2%) + *Fucus spiralis* PSs (0.5%) + *Fucus spiralis* bioactive-rich extract (0.25%) film and the alginate film.

Film	L*	a*	b*	Hue (°)	Chrome
Alginate (3%)	51.79 ± 3.79	2.29 ± 0.15	0.93 ± 0.11	21.86 ± 1.32	2.48 ± 0.18
Alginate (2%) + *F. spiralis* PSs (0.5%) + *F. spiralis* bioactive-rich extract (0.25%)	42.84 ± 6.71	4.07 ± 1.14	10.94 ± 2.74	69.6 ± 0.66	11.67 ± 2.98

**Table 5:** Solubility of the alginate (2%) + *Fucus spiralis* PSs (0.5%) + *Fucus spiralis* bioactive-rich extract (0.25%) film and of the alginate film.

Film	H_2_O	Acetic Acid 3%	EtOH 10%	EtOH 20%	EtOH 50%
Alginate (3%)	100 ± 0.00	16.93 ± 1.65	100 ± 0.00	100 ± 0.00	100 ± 0.00
Alginate (2%) + *F. spiralis* PSs (0.5%) + *F. spiralis* bioactive-rich extract (0.25%)	100 ± 0.00	20.71 ± 6.82	100 ± 0.00	100 ± 0.00	36.12 ± 1.01

**Table 6:** Antioxidant activity (ABTS and DPPH methods) of the films produced.

Film	ABTS	DPPH
	(µM TE/mg Film)	
Alginate (3%)	120.150 ± 6.809	85.967 ± 3.193
Alginate (2%) + *F. spiralis* PSs (0.5%) + *F. spiralis* bioactive-rich extract (0.25%)	221.460 ± 10.389	186.889 ± 36.062

**Table 7:** Nutritional composition of the second alga residue obtained after the extraction of bioactive compounds using MHG, and extraction of polysaccharides.

Component	*Fucus spiralis* Residue	*Fucus* spp. [2]
Water (%)	83.22 ± 0.75	68–88
Ash (g/100 g)	1.56 ± 5.40	19–36
Carbohydrates * (g/100 g)	13.32 ± 9.08	26–66
Protein (g/100 g)	1.50 ± 2.10	1–17
Fat (g/100 g)	0.40 ± 5.70	0.4–5
Fibre: insoluble and soluble	7.86 ± 0.07 1.01 ± 0.02	4–63
Sugar ** (g/100 g)	4.45 ± 4.37	-
Energetic value (kcal)	62.88 ± 1.67	112–377

* Carbohydrates were calculated by difference, subtracting the weight of moisture + protein + ash + fat from the total weight fresh alga. ** Sugars were calculated by difference, subtracting the weight of insoluble and soluble fibre from that of carbohydrates.

**Table 8:** Amino acid profiles present in both *Fucus spiralis* residues (with and without polysaccharides).

Amino Acid	*Fucus spiralis* Residue with PSs (mg/100 mg DW)	*Fucus spiralis* Residue without PSs (mg/100 mg DW)	*Fucus* spp. (g/100 g Protein) [2]
Aspartic acid	3.44 ± 1.31	11.60 ± 0.66	n.a.
Glutamic acid	2.44 ± 0.99	5.62 ± 0.50	9.10–31.47
Cysteine	3.99 ± 0.57	8.22 ± 1.23	n.a.
Asparagine	2.15 ± 0.70	5.74 ± 0.85	7.98–13.85
Histidine	11.49 ± 0.40	22.92 ± 1.31	1.25–1.89
Glycine	10.64 ± 0.04	16.40 ± 0.44	3.63–10.16
Threonine	0.44 ± 0.09	0.37 ± 0.06	2.38–5.97
Arginine	0.69 ± 0.24	1.70 ± 0.14	1.59–4.56
Alanine	n.d.	n.d.	4.93–11.58
Tyrosine	1.89 ± 0.47	3.60 ± 1.24	1.36–2.14
Valine	2.56 ± 0.35	6.89 ± 0.57	3.54–6.50
Methionine	0.67 ± 0.33	1.34 ± 0.05	0.17–4.78
Isoleucine	0.87 ± 0.37	0.99 ± 0.04	n.a.
Leucine	0.82 ± 0.06	1.98 ± 0.48	4.19–7.78

n.a.—not available; n.d.—not detected.

**Table 9:** Mineral contents and oligoelements in both *Fucus spiralis* residues (with PSs and without PSs).

Mineral/Oligoelement	*Fucus spiralis* Residue with PSs (mg/100 g DW)	*Fucus spiralis* Residue without PSs (mg/100 g DW)	*Fucus* spp. (mg/100 g DW) [2]
Zn	1.8 ± 0.1	1.5 ± 0.11	2.6–29
Fe	1.4 ± 0.1	1.0 ± 0.0	4.2–52
Cu	n.d	n.d	0.2–1.4
Na	135.5 ± 8.1	76.5 ± 0.4	630–5469
K	191.0 ± 4.9	119.5 ± 1.1	976–4322
Ca	150.0 ± 5.0	46.5 ± 1.8	118–2175
Mg	68.5 ± 0.5	49.5 ± 1.1	163–994

n.d.—not detected.

## Data Availability

The original contributions presented in the study are included in the article, further inquiries can be directed to the corresponding author.
